# Microbial Primer: Bacterial metallophores – their role for metal homeostasis and social interactions

**DOI:** 10.1099/mic.0.001688

**Published:** 2026-05-05

**Authors:** Clémentine Laffont, Rolf Kümmerli

**Affiliations:** 1 Department of Quantitative Biomedicine, University of Zurich, Winterthurerstrasse 190, 8057 Zürich, Switzerland

**Keywords:** bacterial interactions, clinical and biotechnological applications, metalloproteins, metal toxicity, molecular pathways, secondary metabolites

## Abstract

Metals are essential trace elements for almost all organisms including bacteria. Yet, metals are toxic at high concentrations, requiring fine-tuned regulatory mechanisms to steer metal homeostasis inside cells. In this primer, we explain how bacterial metallophores – small secreted secondary metabolites – act as gatekeepers by carefully orchestrating the scavenging and uptake of essential metals whilst preventing intracellular toxicity and keeping toxic metals outside the cell. We further introduce metallophore diversity together with main synthesis, secretion and uptake mechanisms. Finally, we show how secreted metallophores shape ecological interactions between bacteria and with eukaryotic organisms and how fundamental research on metallophores opens promising avenues for therapeutic and biotechnological applications.

## Metals in biology: essential yet toxic

Metals such as iron, zinc, cobalt or manganese play a crucial role for all life on earth. They are essential micronutrients and serve as cofactors in enzymes, are involved in the folding and stability of proteins and are part of electron transport chains. Thus, they are fundamental to life. It is estimated that nearly 30–50% of proteins involved in metabolism depend on metallic ions for structural integrity and/or catalytic activity. Metals integrated into proteins contribute to a wide array of biological functions, including photosynthesis (magnesium in chlorophyll), oxygen transport (iron in haemoglobin), respiration (iron in cytochrome), immune defence (zinc in matrix metalloproteinase-9), nervous system (copper in dopamine *β*-hydroxylase), protection against reactive oxygen species (ROS) (iron, manganese, nickel, copper or zinc in superoxide dismutase) and metabolism (manganese in glycosyltransferase, cobalt in vitamin B12 and nickel in urease). Among all biologically essential metals, iron and zinc probably stand out in terms of relevance, albeit for different reasons. Iron is emblematic for its ability to alternate between its reduced (Fe²^+^, ferrous) and oxidized (Fe³^+^, ferric) state, a property that underscores its central role in catalytic activities, electron transfer and redox homeostasis. Conversely, zinc only occurs in a single redox state (Zn^2+^), yet it takes on a key role in stabilizing structures of a wide range of proteins from transcription factors to enzymes. These examples highlight the essentiality of metals for cellular physiology across all forms of life.

However, the same elements that sustain life at trace concentrations can become deleterious when present in excess, matching Paracelsus’ famous statement: ‘the dose makes the poison’. Metal toxicity arises through several mechanisms. Elevated intracellular concentration of a specific metal can lead to mismetallation, in which non-cognate metals displace essential cofactors from their binding sites. This typically compromises the catalytic activity of enzymes or the protein’s structural integrity. Moreover, transition metals such as iron and copper can catalyse the formation of ROS such as hydrogen peroxide and hydroxyl radicals. ROS are highly reactive molecules that damage lipids, proteins and nucleic acids in DNA. Persistent oxidative stress can ultimately lead to severe cell damage and death. Overall, metals take on a dual role by being both the architects and the saboteurs of cellular life across all kingdoms including bacteria. To be able to cope with these opposing roles, mechanisms maintaining metal homeostasis are required.

## Metallophores: gatekeepers of bacterial metal homeostasis

**Fig. 1.**
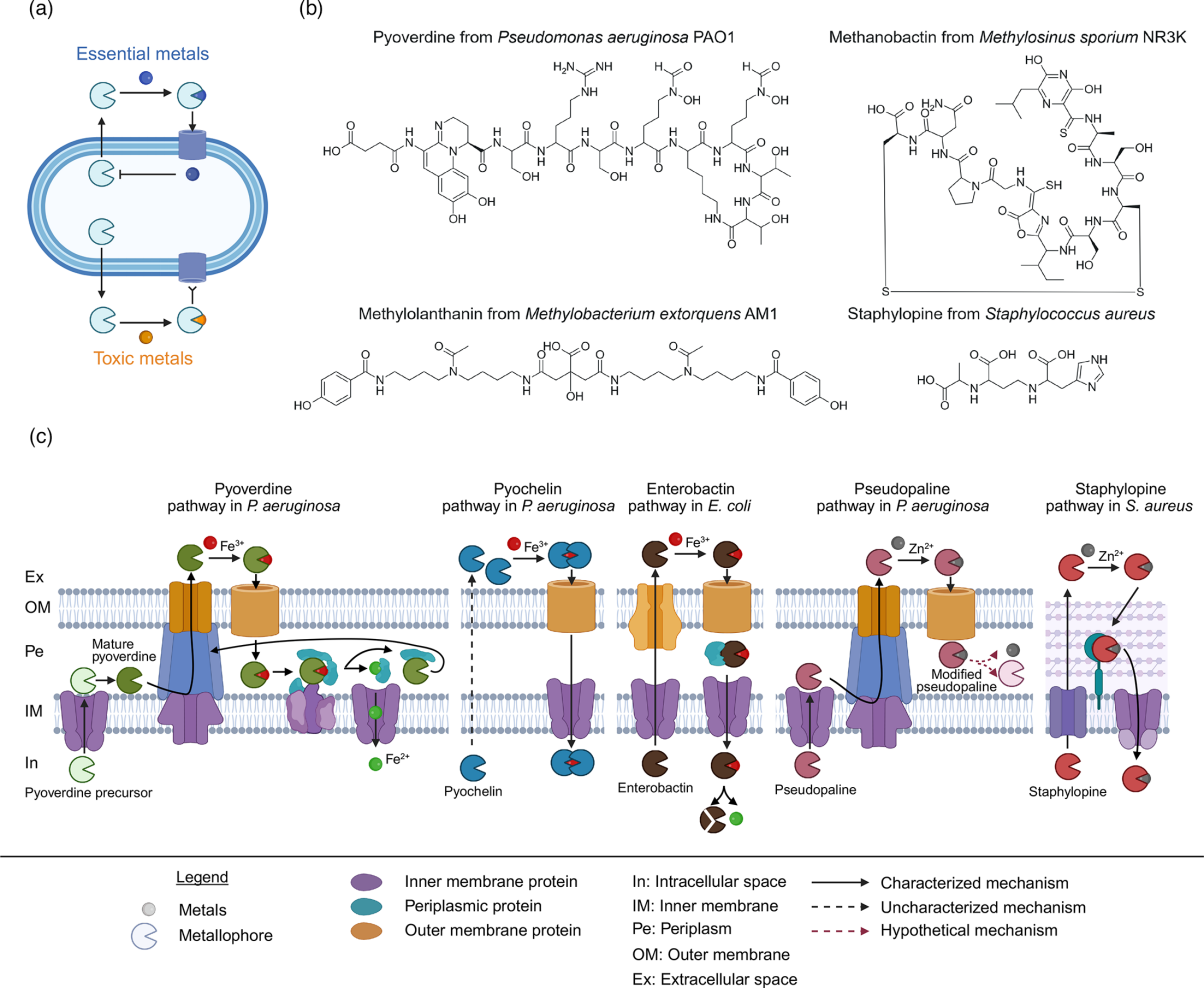
Bacterial metallophores – their function, structural diversity, secretion and uptake mechanisms. (**a**) Schematic showing key functions of bacterial metallophores as gatekeepers of metal homeostasis. Fine-tuned regulatory circuits allow bacteria to sense limitation of essential metals (blue circle), which triggers metallophore production, secretion and uptake. The accumulation of sufficient metal ions within cells leads to the downscaling of metallophore production to minimize the risk of intracellular toxicity. Metallophores bind toxic metals (orange circle). Due to high transporter specificity, the toxic metals cannot enter the cell and remain locked in the environment. High concentrations of toxic metals in the environment can keep metallophore production at a high level. (**b**) Examples of chemical structures of different metallophore types: pyoverdine, siderophore; methanobactin, chalkophore; methylolanthanin, lanthanophore; and staphylopine, nicotianamine-like metallophore. (**c**) Transport pathways of different metallophores in Gram-negative bacteria (siderophores: pyoverdine, pyochelin and enterobactin; nicotianamine-like metallophore: pseudopaline) and Gram-positive bacteria (nicotianamine-like metallophore: staphylopine). All pathways share five steps: (i) intracellular biosynthesis of metallophores, (ii) secretion into the extracellular space through export systems, (iii) metal chelation in the environment, (iv) uptake through specific transport systems, (v) release of metals from metallophore through either metal reduction, hydrolysis or metallophore modification. Created in BioRender. Kümmerli, R. (2025) https://BioRender.com/h7gq5xb.

Metal homeostasis is a fundamental process that maintains the delicate equilibrium between the acquisition of essential metals and the prevention of intracellular toxicity (Fig. 1a). Although metal homeostasis is a universal requirement for all living organisms, this review will focus on bacteria and explain how they balance essential versus toxic effects of metals and which mechanisms of metal homeostasis they deploy. Whilst balancing metal uptake versus toxicity is challenging per se, bacteria frequently encounter the problem that metal bioavailability is low either because metals occur as minerals and are poorly soluble or because they are bound to organic ligands in the environment. Consequently, bacteria need to first make these metals bioavailable. Bacteria solve this challenge through the production and secretion of metallophores. Metallophores are low-molecular-weight metabolites. They are secondary metabolites produced through pathways that are not involved in central metabolism. Metallophores have high affinity for metal ions and are typically secreted into the environment to chelate and solubilize metals from extracellular sources (e.g. minerals or organic ligands). Metallophores complexed with metals are subsequently recognized by specific membrane-transport systems to take up metal ions and feed them either into metabolism or storage. To ensure a steady supply of essential metals but to prevent toxic accumulation of metals inside the cell, bacteria have evolved fine-tuned regulatory mechanisms to sense metal limitation, to control metallophore production levels and to selectively steer metal influx (Fig. 1a).

The high specificity of metallophores for metals and the selectivity of uptake systems are hallmarks of metal homeostasis. There exist many types of metallophores (see next section), and each type has a high binding affinity for the main metal they target. However, since the various metal ions share certain physical properties (e.g. charge and coordination geometry), most metallophores are promiscuous, binding a range of metals although with different affinities. Key components of specificity are the transport systems, which are highly selective and only allow metallophores that are complexed with metabolically essential metals to enter cells. Whilst the scavenging and uptake of metabolically essential metals are recognized as a fundamental function of metallophores, the promiscuous binding of other metals seems to be associated with additional adaptive benefits. Particularly, the binding of toxic heavy metals such as cadmium and lead in polluted environments seems advantageous as the metallophores sequester the toxic metals in the extracellular space and thereby detoxify the environment. There is evidence that bacteria keep metallophore production levels high in environments with high toxic metal concentrations, reinforcing the view that the binding of heavy metals is a secondary function of metallophores. Taken together, the sophisticated properties of metallophores and their uptake systems allow bacteria to selectively scavenge the type and amounts of metals they need for metabolism, whilst minimizing intracellular toxicity and keeping toxic heavy metals outside the cell. Metallophores can thus truly be considered the gatekeepers of metal homeostasis (Fig. 1a).

## Capturing the diversity of metallophores

As already referred to above, there are different types of metallophores (see Fig. 1b for examples). Because metals differ in both their chemical properties and environmental availability, each type of metallophore is optimized to capture specific metal ions under particular ecological conditions. Among others, there are metallophores for iron (siderophores), zinc (zincophores), copper (chalkophores), nickel (nickelophores) and lanthanides (lanthanophores). In addition to these metallophores showing high specificity for particular metals, there also exist molecules that act as generalist scavengers by binding a broad range of metal ions. This is especially the case for members of the nicotianamine-like metallophore family, which can bind multiple divalent metals including iron, zinc, manganese, nickel, copper and cobalt.

Siderophores are by far the most extensively studied group of metallophores. Hundreds of chemically different siderophores have been identified and characterized. Bacterial species (often even strains) differ in the number and types of siderophores they produce. One explanation for the existence of this diversity is ecological competition between species and strains. Given that microbial communities are diverse and individual members compete for the same resources, it is favourable to secrete siderophores that exclusively scavenge iron for members of the same strain or species. In addition to competition, variation in environmental factors also contributes to siderophore diversity. For instance, the highly dilute and diffusive nature of the marine environment seems to select for siderophore types with hydrophobic fatty acid tails that associate with cell membranes and thereby keep siderophores close to producers. Conversely, hydrophilic siderophores are more common among bacterial taxa living in soil or in association with hosts, representing highly structured environments in which diffusion and molecule loss is generally low. Although studied to a lesser extent, chemical diversity also exists for other types of metallophores, and it would be interesting to test whether the same ecological principles (competition and environmental parameters) guide diversity.

## Molecular machineries of metallophore synthesis and uptake

The chemical diversity of metallophores is mirrored in the diversity of pathways involved in their synthesis, secretion and uptake (Fig. 1c). Although the pathways differ widely at the molecular and biochemical level, they all comprise five common steps: (i) the biosynthesis of metallophores within the cell, (ii) the secretion of metallophores into the extracellular environment, (iii) the chelation of the target metal to form a stable complex, (iv) the uptake of the metallophore–metal complex and (v) the intracellular release of the metal. We briefly introduce a few key pathways involved in these steps.

Synthesis: many metallophores are produced through non-ribosomal peptide synthase (NRPS) pathways. NRPSs are large modular enzymes that synthesize small peptides independent of ribosomes. A wide range of amino acids (including non-proteinogenic ones) is used as substrates. Together with tailoring enzymes, NRPS pathways can generate an enormous diversity of secondary metabolites, including metallophores. Other groups of metallophores are produced through NRPS-independent synthesis. These pathways involve enzymes that catalyse the formation of metallophores by assembling metal-chelating moieties (e.g. hydroxamate groups) with variable substrates from central metabolism (e.g. amino acids and citrate).

Secretion: metallophore secretion depends on cell envelope structure and thus differs between Gram-negative and Gram-positive bacteria. In Gram-negative bacteria, metallophore secretion typically involves a two-step process consisting of the translocation of metallophores from the cytosol to the periplasm via specific transporters and their ejection into the extracellular space via efflux pumps. In Gram-positive bacteria, metallophore secretion only requires one transport step as these bacteria possess a single cytoplasmic membrane surrounded by a thick peptidoglycan layer. Dedicated transporters such as those from the major facilitator superfamily mediate the passage.

Chelation: the binding between metal and metallophore is highly dependent on the chemical structure of the metallophore itself as well as the ionic state of the metal. For instance, siderophores have much higher affinity for Fe^3+^ than for Fe^2+^. Moreover, and as mentioned above, some metallophores have high specificity for a single metal (e.g. chalkophores), whilst others exhibit a broader affinity spectrum for multiple metals (e.g. nicotianamine-like metallophores).

Uptake: in Gram-negative bacteria, the uptake of metallophore–metal complexes is guided by TonB-dependent transporters. These transporters consist of a *β*-barrel with a plug domain that controls the selective entrance of metallophores into the periplasm. This process relies on the Ton motor (TonB-ExbB-ExbD complex) using the proton gradient as an energy source. Passage through the cytoplasmic membrane is typically mediated by ABC transporters. In Gram-positive bacteria, metallophore–metal complexes are usually recognized and internalized by specific ABC-transporters composed of substrate-binding proteins (which bind the metallophore–metal complex), a transmembrane domain (which allows the uptake through the membrane) and an ATPase (which provides the required energy).

Metal release: there are three different ways of how bacteria can dissociate metals from metallophores upon internalization. They all involve enzymatic processes that trigger either metal reduction (for redox-active metals such as iron), metallophore hydrolysis or metallophore modification. In Gram-negative bacteria, metal dissociation can occur either in the periplasm or in the cytoplasm depending on the metallophore–metal complex.

## Metallophores drive interactions between bacteria and with hosts

**Fig. 2.**
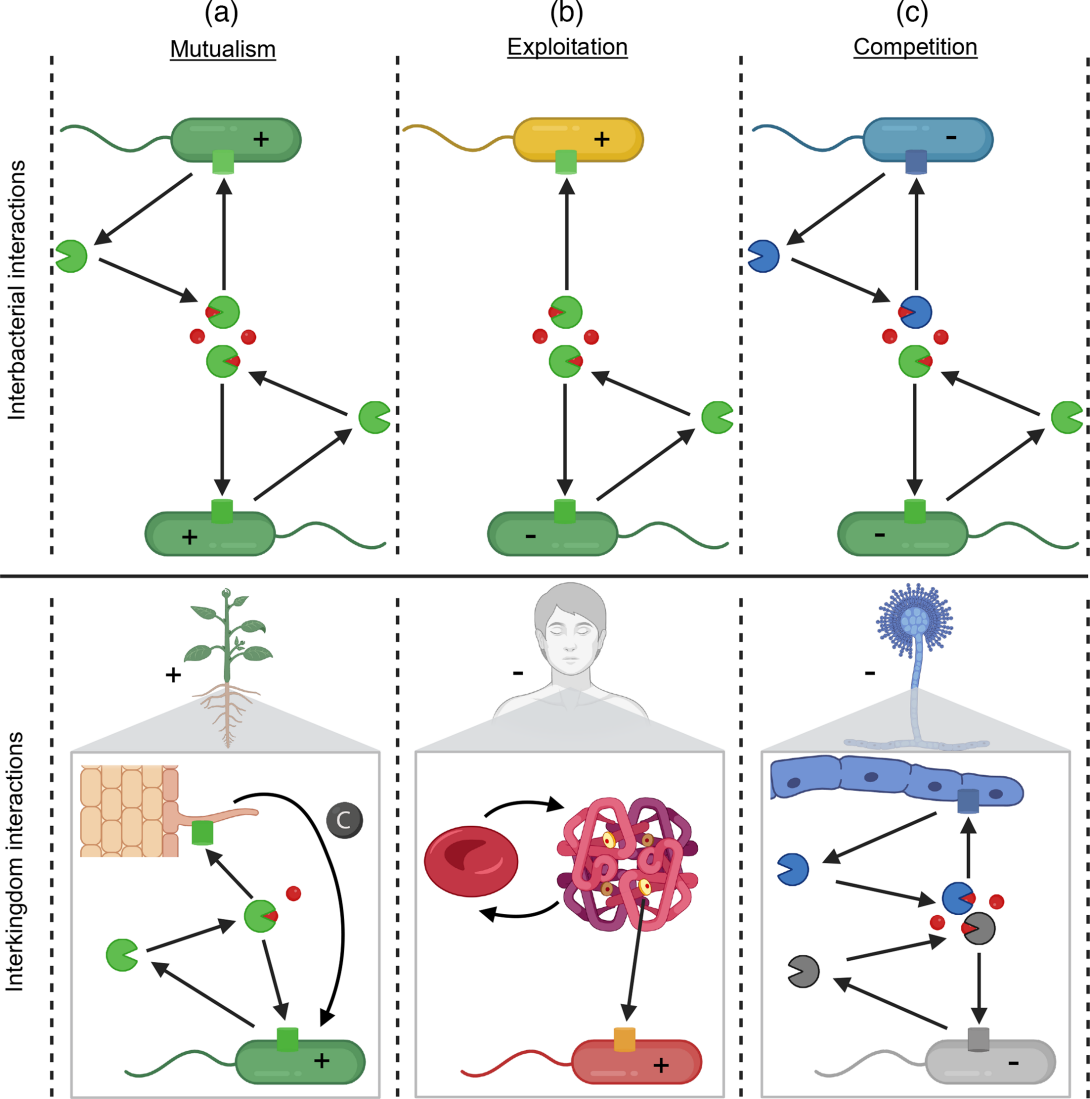
Bacterial metallophores mediate a wide spectrum of ecological interactions, from mutualism to exploitation to competition, both within bacterial communities and in interactions with eukaryotes. Representative examples of social interactions mediated by secreted metallophores (partial circles) that chelate metallic ions (coloured circles) and require specific transporters (cylinders) for uptake. The plus/minus symbols within cells illustrate the fitness effect each species experiences relative to its interaction partner. (**a**) Mutualism occurs when both partners benefit from each other. Among clonally related bacteria, metallophores are shared within a common pool, enhancing collective metal acquisition. Mutualistic interactions can also occur between bacteria and plants: bacterial metallophores facilitate metal uptake not only for bacteria themselves but also for plants equipped with compatible transporter systems, whereas plants reciprocally provide essential nutrients such as carbon sources. (**b**) Exploitation arises when one species benefits at the expense of another. In bacterial communities, exploitation can occur when non-producing bacteria possessing compatible receptors exploit the shared metallophore pool without contributing to it. Interkingdom exploitation can occur when bacterial pathogens target human cells by hijacking haem through specific transporters. (**c**) Competition occurs when both species negatively affect each other. This scenario applies when each species produces specific metallophores that the other one cannot use, thereby restricting the overall metal availability. Such interactions are common between pathogens in polymicrobial infections but also occur in interactions between bacteria and fungi in soil. Created in BioRender. Kümmerli, R. (2025) https://BioRender.com/dj0k48x.

Metallophores are secreted into the environment, and in many situations, the physical connection between producers and molecules is broken. Consequently, cells other than the producers may experience fitness consequences induced by the secreted metallophores. This rationale has led to the discovery that metallophores are important drivers of social interactions between bacteria, but also between bacteria and eukaryotic organisms. Here, we briefly introduce three main interaction types – mutualistic cooperation, exploitation and competition – and illustrate respective examples of how metallophores can drive interactions between bacteria and between bacteria and eukaryotic organisms (Fig. 2).

Mutualistic cooperation (Fig. 2a): metallophores can be shared among cells that all produce and contribute to the common (public) pool of the same type of metallophore. In such a scenario, every individual derives fitness benefits no matter whether it takes up metals chelated to self- or non-self-produced metallophores. Cooperative sharing of metallophores is evolutionarily most stable among clonally related bacteria as their interests are aligned. Conversely, it can also occur among more distantly related strains as long as production (cost) and uptake (benefit) rates are similar among the interacting partners. Since there are many different types of metallophores, there may also be opportunities for the mutual exchange of these metabolites through division of labour between species. At the interkingdom level, mutualistic associations can develop between bacteria and plants, whereby bacterial metallophores facilitate metal acquisition for plants, which in return supply bacteria with essential nutrients such as carbon sources through root exudates.

Exploitation (Fig. 2b): metallophores can be exploited by individuals that express corresponding uptake transporters without contributing to the common metallophore pool themselves. These individuals derive fitness benefits at the cost of producers. Such exploitative behaviours are often termed free-riding, cheating or piracy. Research on siderophores has revealed that bacteria often follow a mixed strategy, by producing and sharing siderophores among close relatives, whilst possessing a diverse array of transporters to exploit (heterologous) siderophores produced by other community members. At the interkingdom level, exploitation often manifests in bacterial pathogens taking up metal-chelating compounds from hosts such as haem–iron and citrate–metal complexes through specific uptake systems. Important to note is that cooperation and exploitation can also occur in the context of metallophore-mediated environmental detoxification. This is because metallophore-producing cells jointly contribute to the detoxification of the environment, whereas non-metallophore-producing cells can free-ride on the service provided by producers.

Competition (Fig. 2c): metallophores can be deployed to prevent competitors from metal scavenging. If all interacting species follow this strategy, then they can mutually inhibit each other’s access to metals and experience negative fitness consequences. Mechanistically, this scenario applies when each species produces a distinct metallophore that the other species cannot use, thereby reducing the overall metal uptake rate in the community. Such competitive (antagonistic) interactions are common among bacterial pathogens in polymicrobial infections but also occur at the interkingdom level, for example, between bacteria and fungi, as well as between bacteria and animal hosts.

## Metallophores and potential applications

Thanks to the mechanistic and ecological insights gained, metallophore systems have become interesting for applied research. Their distinct chemistry and specificity make them promising tools for clinical and technological applications. One line of application makes use of the metallophore-transporter specificity to facilitate the translocation of drugs into bacterial cells by bypassing membrane barriers. In the so-called ‘Trojan horse’ strategy, a metallophore moiety is conjugated to a drug (e.g. antibiotic), which is then taken up by bacteria through their native metallophore transporter system, thereby delivering the drug directly into the cell. Another therapeutic approach consists of the administration of specific metallophores for which pathogens lack compatible uptake systems. Such metallophores deprive pathogens of essential metals, which induces starvation and thereby reduces pathogen growth and survival. With regard to biotechnological applications, metallophores can be used in bioremediation processes. As mentioned before, metallophores can generally bind a broad range of metals including toxic heavy metals. Due to the high transporter specificity, these metallophore–metal complexes will not be internalized but heavy metals remain instead locked in the extracellular space, leading to effective metal sequestration. These properties are harnessed for the design of bioremediation systems that aim to remove toxic heavy metals from polluted environments.

In the end, bacterial metallophores feature key molecular mechanisms to support metal homeostasis in bacteria. Beyond their native roles, these metabolites constitute a powerful and versatile toolbox for promising applications in biotechnological and medical research.

## Further Reading

1. Chandrangsu P, Rensing C, Helmann JD. Metal homeostasis and resistance in bacteria. *Nat Rev Microbiol* 2017;15:338–350. 10.1038/nrmicro.2017.15

2. Jomova K, Makova M, Alomar SY, Alwasel SH, Nepovimova E, *et al*. Essential metals in health and disease. *Chem Biol Interact* 2022;367:110173. 10.1016/j.cbi.2022.110173

3. Maret W. The extracellular metallometabolome: metallophores, metal ionophores, and other chelating agents as natural products. *Nat. Prod. Commun* 2024;19. 10.1177/1934578X241271701

4. Bellotti D, Rowińska-Żyrek M, Remelli M. How zinc-binding systems, expressed by human pathogens, acquire zinc from the colonized host environment: a critical review on zincophores. *Curr Med Chem* 2021;28:7312–7338. 10.2174/1389200222666210514012945

5. Kamyabi G, Debley EL, Nolan EM. Recent advances in metallophore research uncover functions in quorum sensing, antimicrobial activity, and lanthanide acquisition. *Curr Opin Chem Biol* 2025;87:102604. 10.1016/j.cbpa.2025.102604

6. Kenney GE, Rosenzweig AC. Chalkophores. *Annu Rev Biochem* 2018;87:645–676. 10.1146/annurev-biochem-062917-012300

7. Kramer J, Özkaya Ö, Kümmerli R. Bacterial siderophores in community and host interactions. *Nat Rev Microbiol* 2020;18:152–163. 10.1038/s41579-019-0284-4

8. Laffont C, Arnoux P. The ancient roots of nicotianamine: diversity, role, regulation and evolution of nicotianamine-like metallophores. *Metallomics* 2020;12:1480–1493. 10.1039/d0mt00150c

9. Wei Z, Gu S, Vollenweider V, Zuo Y, Li Z, *et al*. Microbial siderophores for one health. *Trends Microbiol* 2025;33:1277–1285. 10.1016/j.tim.2025.05.002

10. Hesse E, O’Brien S, Tromas N, Bayer F, Luján AM, *et al*. Ecological selection of siderophore-producing microbial taxa in response to heavy metal contamination. *Ecol Lett* 2018;21:117–127. 10.1111/ele.12878

## Abbreviations

NRPS: non-ribosomal peptide synthetase
ROS: reactive oxygen species

